# Identifying Novel Candidate Genes Related to Apoptosis from a Protein-Protein Interaction Network

**DOI:** 10.1155/2015/715639

**Published:** 2015-10-04

**Authors:** Baoman Wang, Fei Yuan, Xiangyin Kong, Lan-Dian Hu, Yu-Dong Cai

**Affiliations:** ^1^Institute of Health Sciences, Shanghai Institutes for Biological Sciences, Chinese Academy of Sciences, Shanghai 200031, China; ^2^College of Life Science, Shanghai University, Shanghai 200444, China

## Abstract

Apoptosis is the process of programmed cell death (PCD) that occurs in multicellular organisms. This process of normal cell death is required to maintain the balance of homeostasis. In addition, some diseases, such as obesity, cancer, and neurodegenerative diseases, can be cured through apoptosis, which produces few side effects. An effective comprehension of the mechanisms underlying apoptosis will be helpful to prevent and treat some diseases. The identification of genes related to apoptosis is essential to uncover its underlying mechanisms. In this study, a computational method was proposed to identify novel candidate genes related to apoptosis. First, protein-protein interaction information was used to construct a weighted graph. Second, a shortest path algorithm was applied to the graph to search for new candidate genes. Finally, the obtained genes were filtered by a permutation test. As a result, 26 genes were obtained, and we discuss their likelihood of being novel apoptosis-related genes by collecting evidence from published literature.

## 1. Introduction

Apoptosis, an efficient cell death program, plays an important role in maintaining strictly regulated organismal homeostasis and involves the interaction of multiple factors. Since the mid-nineteenth century, cell death has been widely studied, and researchers have learned that all physiological processes of multicellular organisms involve cell death, particularly during embryogenesis and metamorphosis [1, 2]. The first, the second, and the third PCD are the primary forms of apoptosis. The well-known caspase-dependent apoptosis is the first PCD. During the process of the second PCD, some vacuoles appear that have two membranes and autophagy functions; however, we know little regarding the third PCD. The second and the third PCD belong to caspase-independent apoptosis [3]. In the first, second, or third PCD, apoptosis maintains organism homeostasis and helps organism survival by defending against exogenous or endogenous toxic compounds. The intrinsic and extrinsic pathways have been well studied as the typical apoptotic processes [4–6]. Activated cell surface receptors mediate extrinsic apoptosis and transmit apoptotic signals through the combination of receptors and ligands. Death receptors consist of the tumor necrosis factor receptor gene superfamily, such as* TNFR-1*,* Fas/CD95*, and the* TRAIL* receptors* DR-4* and* DR-5* [7]. The first type PCD cells can bring about caspase-dependent apoptosis pathways [8]. A caspase cascade that is extreme enough to execute cell death cannot be generated from activated receptors in the second type PCD cells, and the signal amplification depends on mitochondria-dependent apoptotic pathways. Mitochondria, which are the central regulator of intrinsic apoptosis pathways and communicate with organelles, can connect the different apoptosis pathways [4]. The apoptosis pathway also involves some ion channels. The calcium channel represents the typical ion channel, and calcium ion concentration in the cytosol participates in signal transduction, cell death, and proliferation. Moreover, calcium channel opening or closing controls cell fate.

Organisms regulate their development and maintain through sophisticated interplay between cells. During development, organisms produce excess cells that finally go through PCD and contribute to the formation of organic structures [9]. In interdigital mesenchymal tissue, the formation of independent digits through massive cell death is a typical example of PCD in development [10]. Apoptosis processes possess great biological significance, being involved in differentiation, development, proliferation, regulation, and so forth. Therefore, a variety of pathological conditions present dysregulation or dysfunction of the apoptotic program. Disorders in apoptosis can induce cancer, viral infection, and autoimmune disease; however, abnormal apoptosis will induce AIDS and neurodegenerative disease [11]. Multiple internal and external stimuli, such as ligands binding cell surface receptors, treatment with cytotoxic drugs or irradiation, DNA damage, contradictory cell cycle signaling, death signals, or a lack of survival signals can trigger apoptosis. The initiation, mediation, or execution of apoptosis involves many factors and once the genes encoding these factors mutate, the death machinery can be dysfunctional. Moreover, researchers have found that several mutations in apoptosis genes induce human diseases as initiating or contributing factors [12]. The excessive proliferation induced by the activation of oncogenes and disorders in apoptosis checkpoints have become primary factors in tumorigenesis over the last years [13].

Apoptosis contributes to maintaining the balance of homeostasis by normal cell death [3]. Necrosis can result in inflammation, but apoptosis yields few side effects. As such, apoptosis can be therapeutic targets to treat some diseases, for example, obesity [14], cancer, and neurodegenerative diseases. Therefore, the identification of key apoptosis-related genes before disease occurrence will greatly help in the prevention and treatment of disease. However, it is inefficient to discover novel apoptosis-related genes using traditional experiments. Building effective computational methods can highly increase this efficiency. We therefore proposed a computational method to identify apoptosis-related genes in this study. Twenty-six new genes were identified, which were related to the biological processes of apoptosis by analyzing previously published literature.

## 2. Materials and Methods

### 2.1. Genes Related to Apoptosis

Previously known apoptosis-related genes were obtained from KEGG [15], a database resource for understanding high-level functions and utilities of biological systems from molecular-level information. In detail, 86 human genes were extracted from the information in the pathway hsa04210: Apoptosis-Homo sapiens (human) from the website: http://www.genome.jp/dbget-bin/www_bget?hsa04210. The names of these genes are available in Supplementary Material I available online at http://dx.doi.org/10.1155/2015/715639.

### 2.2. Method to Identify Novel Candidate Genes

To identify the novel candidate genes related to apoptosis, we used protein-protein interaction information that was retrieved from the STRING (Search Tool for the Retrieval of Interacting Genes/Proteins, Version 9.0, http://www.string-db.org/) database [16] to construct a weighted graph. The weighted graph's construction procedures were the same as those in [17–19]. Here, we gave the brief description of these procedures; readers can refer to these studies for additional details. From the obtained file (protein.links.v9.0.txt.gz) retrieved from STRING, we extracted all protein-protein interactions of human. Each extracted protein-protein interaction of human consists of two proteins, represented by Ensembl IDs, and one score that evaluates the strength of the interaction with range between 150 and 999. The constructed graph took proteins, collected from all obtained protein-protein interactions of humans, as nodes and two nodes were adjacent if and only if the corresponding proteins can comprise an interaction. Obviously, each edge represented a protein-protein interaction of human. In addition, each edge was assigned a weight, which was defined as 1,000 minus the interaction score of the corresponding interaction.

The shortest path algorithm, Dijkstra's algorithm [20], was executed on the constructed graph to search for the shortest paths connecting any two known apoptosis-related genes. According to the definition of edge weight, consecutive genes in a shortest path were in an interaction with high interaction score, meaning they are more likely to share similar functions. The obtained shortest paths were used to calculate the betweenness of each node/gene in the constructed graph, which is defined as the number of shortest paths containing a certain node/gene as an inner node. Then we excluded genes with betweenness equal to zero and apoptosis-related genes. The remaining genes were further filtered with a permutation test. 500 gene sets were produced by randomly selecting genes in the constructed graph and these gene sets had the same sizes of the apoptosis-related gene set. For each gene set, all shortest paths connecting any two genes in the set were searched in the graph. The betweenness for each remaining genes was calculated based on these paths. Accordingly, for each remaining gene, there were 500 betweenness on 500 randomly produced sets and one betweenness on the apoptosis-related gene set. Another measurement, permutation FDR, was calculated for each remaining gene, which was defined as the ratio of the number of randomly produced gene sets in which the betweenness was larger than that of the known apoptosis-related gene set and the total number of randomly produced gene sets (500). Genes with permutation FDRs less than 0.05 were finally selected as significantly associated with apoptosis.

## 3. Results and Discussions

**Figure 1:**
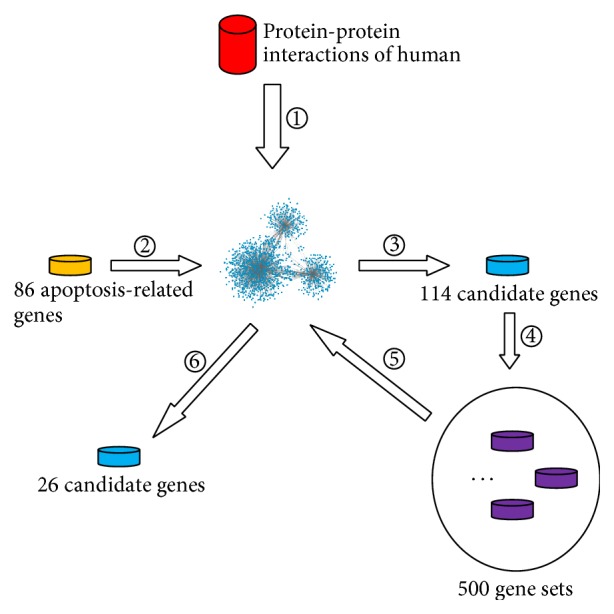
The procedure and result of each step in our method. ① Protein-protein interactions of human were used to construct a graph; ② all shortest paths connecting 86 known apoptosis-related genes were searched in the constructed graph; ③ 114 candidate genes were extracted from the obtained shortest paths; ④ 500 gene sets were randomly produced to execute permutation test; ⑤ for each gene set, all shortest paths connecting genes in the set were searched in the constructed graph; ⑥ 26 candidate genes were finally obtained by calculating permutation FDR.

Based on 86 known apoptosis-related genes, some candidate genes can be obtained according to the method described in Section 2.2. The detailed procedure and result of each step is illustrated in Figure 1.

### 3.1. Results of the Method

The shortest paths connecting any pair of the 86 human genes related to apoptosis were searched in a constructed weighted graph. We discovered 114 genes with betweenness greater than zero (Supplementary Material II). Additionally, a permutation test excluded false discoveries that had high betweenness and little relationship with apoptosis by calculating permutation FDR for each of 114 candidate genes and setting 0.05 as the threshold. We finally obtained 26 genes, which are listed in Table 1. These genes are termed as significant candidate genes in the remaining parts of the paper.

### 3.2. Analysis of Significant Candidate Genes

We finally obtained 26 significant candidate genes for apoptosis. The following paragraphs gave detailed discussion on the relationships between these genes and apoptosis.

#### 3.2.1. *TRAF6* and* TNFRSF1B*

*TRAF6* (betweenness: 509, permutation FDR: <0.002; refer to Table 1, row 1) possesses unique receptor-binding specificity, which is vital as the signaling mediator in TNF receptor superfamily and the IL-1R/Toll-like receptor superfamily signaling pathway [21]. Because of its central convergence in different signal pathways,* TRAF6* is involved in regulating cell death, survival, and cellular responses to stress. Ample studies have shown that TRAF6 is involved in various cell apoptosis conditions. Most of these studies have suggested that TRAF6 regulates cell apoptosis by mediating the caspase-associated signaling pathway. In summary,* TRAF6* acts as a bifurcation point of the survival and death pathways. The subtle regulation by* TRAF6* in the imbalance of survival and death will finally determine cell fate and be a therapeutic target. In our study, one of these genes,* TNFRSF1B* (*TNFR2*) (betweenness: 86, permutation FDR: <0.002; refer to Table 1, row 2), belongs to the TNF receptor superfamily. For a long time, we have known little regarding TNF-induced signaling through TNFRSF1B and the mechanism of TNFR2-mediated cell death. A previous study demonstrated that TNFR2 triggers cell death in the presence of RIP, whereas without RIP [22] NF-*κ*B is activated by TNFR2. Recently, researchers have identified that TNFRSF1B communicates with the JNK and TNF-induced NF-*κ*B signaling pathways in vascular endothelial cells (EC). Understanding the TNFR2-mediated apoptotic and JNK signaling pathways may offer novel therapeutic targets to treat vascular diseases in EC [23]. This observation gives us more confidence in the accuracy of our calculation method. From the above description, we know that* TRAF6* plays an important role in the signaling mediated by the TNF receptor superfamily. Therefore, we hypothesize that these two genes may have synergistic effects on apoptosis regulation, which requires further validation.

#### 3.2.2. *IQGAP1*

*IQGAP1* (betweenness: 289, permutation FDR: <0.002; refer to Table 1, row 3) belongs to a member of the* IQGAP* family and induces multiple cellular functions by interacting with its target proteins. Previous studies have found that IQGAP1 has interactions not only with cell adhesion molecules but also with cytoskeletal components and integrates multiple signaling pathways to regulate cell morphology and motility. Compared with normal tissue, IQGAP1 is overexpressed in colorectal carcinoma [24–26], breast cancer [26], astrocytoma, and head and neck squamous cell carcinoma [27], enhancing cell proliferation, migration, and invasion. Recently, research has indicated that it is also closely related to cell survival and apoptosis. ERK plays a vital role in several biological processes, particularly those involving cellular proliferation, differentiation, survival, and apoptosis [28]. In a mouse model of cardiac hypertrophy, IQGAP1 regulates Melusin-dependent cardiomyocyte hypertrophy and apoptosis via activation of MEK/ERK [29]. In addition, the interaction between RNase L and IQGAP1 can promote ECyd-induced apoptosis [30]. Taken together, we can speculate that* IQGAP1* plays a vital role in apoptosis and survival through signaling pathways, such as the MEK/ERK pathways and their partner proteins.

#### 3.2.3. *FURIN*

*FURIN* (betweenness: 252, permutation FDR: <0.002; refer to Table 1, row 4) is a cellular endoprotease and participates in embryo formation and the maturation of proprotein substrates, which includes extra-cellular-matrix proteins, receptors, and other protease systems. Few studies have reported a direct relationship between* FURIN* and apoptosis, but Yang et al. have recently suggested that* FURIN* may be involved in regulating the proliferation and apoptosis of granulosa cells, because after* FURIN* was knocked down, the apoptosis of the granulosa cells was significantly increased from large antral/preovulatory follicles through downregulation of the antiapoptotic proteins XIAP and p-AKT [31]. Moreover, FURIN can activate massive proprotein substrates and is ubiquitously expressed and participates in many physiological and pathological processes. In our study, because* FURIN* displayed a higher betweenness value, we speculate that FURIN may indirectly be involved in the regulation of apoptosis through activating proprotein substrates in some signaling pathways.

#### 3.2.4. *NFATC1* and* NFATC2*

NFATC1 (betweenness: 95, permutation FDR: <0.002; refer to Table 1, row 5) and NFATC2 (betweenness: 238, permutation FDR: <0.002; refer to Table 1, row 6) are the most famous NFAT factors in peripheral T cells and have similar function but different modes of expression.* NFATC2* belongs to the nuclear factor of activated T cells family and is a transcription factor involved in differentiation in lymphocytes. Many studies have demonstrated that* NFATC2* is involved in the regulation of apoptosis. In a* NFATC2*−/− mice model,* NFATC2*−/− cells not only presented an increase in apoptosis but also presented hyperproliferation [32]. Researchers have demonstrated that overexpression or activation of* NFAT1* can induce cell death in different cell types, for example, T lymphocytes, Burkitt's lymphoma, megakaryocytes, and fibroblasts [33–35]. Moreover, the calcineurin/NFATC2 pathway has an antiapoptotic role in melanoma cells. Apoptosis is induced by NFAT1 through cooperation with the Ras/Raf/MEK/ERK pathway and upregulates TNF-*α* expression in NIH3T3 fibroblasts [36]. Overexpression of* NFATC1* increases* TRAIL* expression in HT29 and Caco-2 cells and also induces FasL [37] and TNF-*α* expression upregulation in several cell types. For some time, the members of* NFAT* family have been considered to be redundant proteins. Nevertheless, in the regulation of cell proliferation and apoptosis, different roles for the* NFAT* family were identified by analyzing mice deficient for NFAT proteins. As transcription factors, the promoter regions of diverse activation-inducible genes all contain binding sites for NFAT proteins [32]. These activation-inducible genes include cytokines IL-2, IL-4, IL-5, and IFN-*γ* and cell surface proteins [33, 38, 39], suggesting that these transcription factors may participate in controlling the cell cycle and apoptosis [40, 41].

Constitutively active NFAT1 (CA-NFAT1) and NFAT2 short isoform (CA-NFAT2/A) mutants localize in the nucleus, bind DNA with high affinity, and activate endogenous* NFAT* target genes [34, 42]. Remarkably, in cell apoptosis, cycle, and transformation regulation, the abnormal expression of the CA-NFAT1 and CA-NFAT2 short isoform in NIH 3T3 fibroblasts presented opposite phenotypes. The NFAT2 short isoform acted as a repressor of cell death and a positive regulator of cell proliferation. Conversely,* NFAT1* increased cell death and repressed the cell cycle. In summary, the* NFAT1* and* NFAT2* genes present opposing roles in regulation of the cell cycle and apoptosis. Moreover, the* NFAT1* and* NFAT2* short isoform genes play dual roles as tumor suppressor or oncogene. The cell phenotype was transformed by CA-NFAT2; however, CA-NFAT1 could suppress the transformation, suggesting that different family members might have complementary functions, and the complementary functions might determine whether the cell lives or dies. This observation also suggests that the cellular threshold levels of each NFAT protein and protein isoform determine the expression of a particular set of target genes, and ultimately, this process determines the fate of the cell. However, more work is necessary to help us better understand the physiological role of the balance between the NFAT1 and NFAT2 short isoforms.

#### 3.2.5. *AKAP5*

Betweenness of this gene was 195 and its permutation FDR was <0.002 (refer to Table 1, row 7). A-kinase anchoring proteins (AKAPs) mediate the localization of the c-AMP-dependent protein kinase (PKA) and other signaling enzymes. No studies have yet indicated that* AKAP5* is directly related to apoptosis. We hypothesize that* AKAP5* may be involved in cell apoptosis by forming complexes with protein kinases, phosphatases, or scaffold proteins. The assembly and localization of signaling complexes are coordinated by scaffold, anchoring, and adaptor proteins to provide efficiency and specificity in signal transduction [43]. It therefore seems reasonable that defects in anchoring protein genes or pathophysiological changes in AKAP signaling complexes may underlie certain damages in cells or tissues. Studies have shown that AKAP5 can form a complex with IQGAP1, and the complex also contributes to the c-AMP/PKA signaling pathway. Because IQGAP1 is a scaffold protein and is involved in apoptosis regulation, it is not surprising that* AKAP5* is also linked to apoptosis. In addition, AKAP5 can interact with ADCY8 to regulate Ca^2+^-dependent c-AMP synthesis in pancreatic and neuronal systems [44]. In the calcium signaling pathway, ADCY8 catalyzes the formation of c-AMP, which phosphorylates PKA to induce the endoplasmic reticulum to release Ca^2+^, resulting in the expression of genes such as* CALM* and* CAMK* that cause cell proliferation and apoptosis. PKA also inhibits phosphorylation of BAD and suppresses apoptosis. Further experimental verification is necessary to test these hypotheses.

#### 3.2.6. *DIABLO*

*DIABLO* (betweenness: 138, permutation FDR: <0.002; refer to Table 1, row 8), also called* Smac*, is a factor that has been shown to exit mitochondria in response to apoptotic stimuli and potentiate caspase activity. The function of* DIABLO* has been elaborated in detail. The inhibitory effect on both initiator and effector caspases is relieved by Smac through interacting with multiple IAPs [45–48], finally promoting apoptosis. Therefore,* Smac/DIABLO* may play a significant role in diagnostic and therapeutic features in cancer. Increasing data suggests that chemoradiation-resistance to apoptosis may result from decreased levels of Smac/DIABLO in advanced colon cancer [49]. In addition, numerous studies have observed that Smac mRNA expression is significantly lower in melanoma, prostate cancer, lung cancer, gastric cancer, colon cancer, and so forth [50–52]. Therefore, the design and development of small-molecule Smac mimetics as novel therapy targets is promising.

#### 3.2.7. *TAB1*

The betweenness of this gene was 120 and its permutation FDR was <0.002 (refer to Table 1, row 9). The TAB1 protein is a regulator of the MAP kinase kinase kinase MAP3K7/TAK1 and can mediate various intracellular signaling pathways. This protein interacts and activates TAK1 kinase. TAK1 mediates multiple inducible transcription factors, such as NF-*κ*B and JNKs [53], which contribute to the development of the embryo, cell survival, and innate immunity. The inhibition of TAK1 activity will suppress cancer cell death, and TAB1 interacts with TAK1 and promotes its autophosphorylation. The interaction between TAB1 and TAK1 therefore controls biological processes, particularly apoptosis. Research has also demonstrated that greater amounts of Xenopus* TAB1* (*xTAB1*) and* xTAK1* mRNAs injected into early embryos can result in cell death [54]. Many investigators have reported that XIAP not only functionally interacts with the BMP receptor but also with the adapter molecule TAB1, and in the presence of the transforming growth factor *β*1 (TGF-*β*1), TAK1 activates JNK1 and p38 as an upstream MAP3 kinase. The XIAP/TAK1-mediated activation of JNK1 depends on TAB1, and the XIAP/TAK1-mediated activation of JNK1 is involved in protection against apoptosis [55]. The proapoptotic pathway TAB1/p38 also mediates apoptosis [56]. In TRAIL-induced apoptotic pathways, the blockade of TAB1 activity enhances apoptosis through the activation of a caspase cascade. In addition, the BIR1 (a domain of XIAP)/TAB1 interaction is crucial for XIAP-induced NF-*κ*B activation [57]. Taken together, we find that* TAB1* plays a vital role in regulating apoptosis and survival, consistent with our expectation.

#### 3.2.8. *CAPNS1* and* CAPN3*

CAPNS1 (betweenness: 86, permutation FDR: <0.002; refer to Table 1, row 10), as a common small regulatory subunit of calpains, is required to maintain the stability and activity of calpains. Some studies have reported that the BCL-2, procaspase 3, and Bax families are all calpain substrates and have confirmed a role for calpain during B and T cell development and apoptosis [58–60]. Because CAPNS1 is a common small regulatory subunit of calpains and contributes to maintaining the stability and activity of calpains, we presume that* CAPNS1* may indirectly be involved in the regulation of apoptosis. In addition, some recent studies have demonstrated that CAPNS1 participates in signaling pathways as a partner. In the Ras signaling pathway, CAPNS1 binds the RasGAP-SH3 domain in* K-Ras* (V12) oncogenic cells, modulating migration and cell survival, and the interaction between CAPNS1 and PP2A-Akt affects FoxO3A-dependent cell death [61]. In addition, calpain 3 belongs to the calpain family of calcium-dependent intracellular proteases, which also plays important roles in regulating apoptosis. The generation of the limb-girdle muscular dystrophy type 2A (LGMD2A) involves* CAPNS1* mutation. The muscular biopsy specimens of LGMD2A patients show that lack of calpain 3 causes I*κ*B*α* accumulation and prevents NF-*κ*B nuclear translocation, ultimately resulting in apoptosis. Moreover, deficiency in CAPN3 (betweenness: 86, permutation FDR: <0.002; refer to Table 1, row 11) is also associated with downregulation of the antiapoptotic factor c-FLIP and myonuclear apoptosis in LGMD2A muscles [62]. Whether* CAPNS1* and* CAPN3* interact or coordinately regulate apoptosis still must be studied.

#### 3.2.9. *ADCY8*

The betweenness of this gene was 167 and its permutation FDR was 0.002 (refer to Table 1, row 12). Adenylate cyclase is a membrane-bound enzyme that contributes to the formation of cyclic AMP from ATP. Although no research has identified its direct relationship with apoptosis, it is an important member of the calcium signaling pathway. In the c-AMP signaling pathway, ADCY8 catalyzes the formation of c-AMP, which in turn induces the activation of PKA. PKA can promote the expression of many genes and influence the endoplasmic reticulum in regulating calcium concentration. In the regulation of signal transduction, the concentration of calcium in the cytosol plays a vital role and is involved in cell death and proliferation. In addition, calcium also can trigger cytochrome c release that does not depend on Bcl-2. In addition to its involvement in apoptosis, the calcium ion is also involved in many other signal pathways by controlling the ion channel's opening and closing [3]. Therefore, we presume that* ADCY8* may serve as a bridge between extracellular stimuli and apoptosis.

#### 3.2.10. *NPRL3*

The betweenness of this gene was 83 and its permutation FDR was 0.008 (refer to Table 1, row 13). So far, we know little about the function of the encoded protein of NPRL3. However, its homolog NPR3 has been recently investigated in yeast and Drosophila [63]. Studies in yeast have demonstrated that an amino acid starvation signal to the target of rapamycin complex 1 (TORC1) can be mediated by the Npr2/3 complex, and artificially inhibiting TORC1 by rapamycin can rescue proliferation defects observed in npr2Δ and npr3Δ cells [63]. In addition, a study has demonstrated that in the female germ line in Drosophila TORC1 signaling can be inhibited by NPRL2 and NPRL3 in the absence of amino acids. In young egg chambers, apoptosis is inhibited by NPRL2 and NPRL3 by downregulating TORC1 activity in the condition of lack of nutrients. In addition, TORC1 is a key regulator of cell growth in response to amino acid availability [64]. Thus, these data suggest that TORC1 activity remains particularly high during periods of amino acid scarcity or other stress circumstances, and subsequently a cell death program will be initiated by a metabolic checkpoint. Nevertheless, the role of* NPRL3* in apoptosis in humans requires further research.

#### 3.2.11. *SMAD*

The betweenness of this gene was 25 and its permutation FDR was 0.014 (refer to Table 1, row 14). The SMAD6 protein is a member of the* SMAD* family. SMAD proteins can mediate multiple signaling pathways through their roles as signal transducers and transcriptional modulators and negatively regulate BMP and TGF-beta/activin-signaling. Members of the TGF-beta family regulate multiple cellular processes, including cell proliferation, differentiation, organization, migration, and death. In addition, SMAD6 and SMAD2 predict overall survival in oral squamous cell carcinoma patients. However, the role of aberrant TGF-beta signaling is not clear [65]. In addition, in the lung adenocarcinoma cell line H1299, knockdown of* SMAD6* upregulates the plasminogen activator inhibitor-1 and phosphorylates SMAD2/3, finally activating TGF-beta signaling. Furthermore, because of the* SMAD6* knockdown, the JNK pathway is also activated and the phosphorylation of Rb-1 is reduced, causing G0-G1 cell apoptosis and arrest [66]. According to the above description, SMAD6 is a key factor in lung cancer cell growth and survival. Therefore, targeted inactivation of SMAD6 may open a new road for treating lung cancer. Moreover, when some lymphoma cell lines were exposed to TGF-*β*, Bcl-xl and Bcl-2 were downregulated, whereas Bax was upregulated. Furthermore, the mRNAs of* SMAD6* and* SMAD7* displayed significant upregulation [67]. These results indicated that the induction of apoptotic pathways may depend on alteration of the gene expression and protein levels. Another study has demonstrated that the TRAF6-TAK1-p38 MAPK/JNK pathway, a noncanonical TGF-*β* pathway, can be induced by TGF-*β*1; however, this process can be negatively regulated through the SMAD6 but not SMAD7. K63-linked poly-ubiquitination of TRAF6 can be abolished through the TGF-*β*1-induced SMAD6 in primary hepatocytes and AML-12 mouse liver cells. In addition, in cell culture or animal models, phosphorylation of TAK1 and p38 MAPK/JNK is maintained and apoptosis increased after knockdown of SMAD6 or A29, suggesting an important role of the SMAD6-A20 axis in negative regulation of the TGF-*β*1-TRAF6-TAK1-p38 MAPK/JNK pathway [68]. Recent research has shown that galangin can induce autophagy by activating the TGF-*β* receptor/SMAD pathway in HepG2 cells. In this process, SMAD6 and SMAD7 expression levels both decreased [69]. Taken together, SMAD6, as a negative regulator, participates in TGF-beta mediated apoptosis.

#### 3.2.12. *IRS1*

The betweenness of this gene was 238 and its permutation FDR was 0.016 (refer to Table 1, row 15). The insulin receptor tyrosine kinase can phosphorylate the IRS1 protein. This gene mutates in type II diabetes with susceptibleness to insulin resistance. IRS1, as a member of the PI3K/AKT signaling pathway, regulates cell survival and apoptosis. A common Arg972 polymorphism in* IRS-1* affects the PI3-kinase/Akt survival pathway, which in turn results in resistance to the antiapoptotic effects of insulin. In addition, the Arg972 polymorphism also impairs human *β*-cell survival [70]. A report has observed that PTPL1 dephosphorylates IRS1, and PTPL1 expression can block the IRS-1/PI3K/Akt signaling pathway [71], finally inhibiting the insulin-like growth factor-I effect on cell survival and apoptosis.

#### 3.2.13. *PKD2*

The protein encoded by* PKD2* (betweenness: 3, permutation FDR: 0.016; refer to Table 1, row 16) is a transmembrane protein and a calcium-permeable cation channel. In addition,* PKD2* is also responsible for transporting calcium signaling in renal epithelial cells. Calcium concentration changes can induce a series of cell biology processes to occur, such as activation of the MAPK signaling pathway [72], eventually controlling cell survival and apoptosis. In addition, calcium also activates the JNK pathway, which can subsequently stimulate Bax activation [3]. Taken together, we speculate that* PKD2* may regulate calcium cations through opening and closing calcium channels, thereby triggering physiological and pathological change and finally deciding cell fate.

#### 3.2.14. *CSF2*

The protein encoded by* CSF2* (betweenness: 65, permutation FDR: 0.02; refer to Table 1, row 17) is a cytokine that regulates the differentiation and function of granulocytes and macrophages. Recently, some studies have reported that* CSF2* is associated with apoptosis. CSF2 can block apoptosis in bovine embryos through interaction with genes controlling apoptosis [73]. In addition, in advanced atherosclerosis,* GM-CSF* promotes macrophage apoptosis and plaque necrosis through IL-23 signaling [74].

#### 3.2.15. *BBC3* (*PUMA*)

*BBC3* (betweenness: 8, permutation FDR: 0.02; refer to Table 1, row 18) belongs to a member of the* BCL-2* family. These family members are also in the BH3-only proapoptotic subclass. This protein induces mitochondrial outer membrane permeabilization and apoptosis through cooperating with direct activator proteins. As mentioned above, DIABLO is released from mitochondria, which can be increased through mitochondrial outer membrane permeabilization enhancement. In addition,* BBC3* was identified 12 years ago, mediates p53-dependent and p53-independent apoptosis, and is also involved in the intrinsic apoptosis pathway [75]. In the induction of apoptosis, a key regulatory step is PUMA binding to the inhibitory members of the Bcl-2 family (Bcl-2-like proteins), such as Bax/Bak, via its BH3 domain, which induces Smac/DIABLO release from mitochondria, finally resulting in intrinsic apoptosis [76].

#### 3.2.16. *NOTCH1*

The betweenness of this gene was 249 and its permutation FDR was 0.022 (refer to Table 1, row 19).* NOTCH1* encodes a member of the NOTCH family. NOTCH signaling participates in maintaining the balance of cell proliferation, differentiation, and apoptosis; therefore, disorders in NOTCH signaling may induce tumorigenesis. The active form of NOTCH1, NOTCH1-ICN, is involved in many cell processes, such as T/B cell development and apoptosis, progression, and deterioration of various cancers. NOTCH1 induces resistance to glucocorticoid-induced apoptosis in developing thymocytes through downregulation of SRG3 expression [77]. In addition, downregulated expression of NOTCH1 promotes apoptosis and cell growth inhibition in pancreatic cancer cells [78]. Apoptosis is induced and cell proliferation is inhibited after the NOTCH1 signaling pathway is activated in the human esophageal squamous cell carcinoma cell line EC9706 [79]. Moreover, NOTCH1 also regulates apoptosis through participating in survival and apoptosis pathways. For example, NOTCH1 signaling can inhibit Akt/Hdm2-mediated p53 degradation and sensitizes human hepatocellular carcinoma (HCC) cells to TRAIL-induced apoptosis. NOTCH1 also inhibits apoptosis through activation of the PI3K-PKB/Akt pathway [80, 81].

#### 3.2.17. *PTPN1* (*PTP1B*)

The protein encoded by* PTPN1* (betweenness: 93, permutation FDR: 0.024; refer to Table 1, row 20) belongs to the protein tyrosine phosphatase (PTP) family. PTPs have been well-known to regulate many cellular events, such as cell growth, differentiation, motility, and proliferation [82]. PTP-1B can also regulate the phosphorylation status of apoptotic proteins [83]. Therefore, research suggests that the apoptosis of hepatocytes caused by serum withdrawal can be protected through PTP1B deficiency [84], whereas its overexpression increases cellular events and results in apoptotic cell death. In cardiomyocytes, hypoxia/reoxygenation-induced apoptosis is also reduced by siRNA targeted to* PTP1B*. PTP1B deficiency is also involved in protecting against Fas-induced hepatic failure [85].

#### 3.2.18. *MAF* (*c-MAF*)

The betweenness of this gene was 54 and its permutation FDR was 0.026 (refer to Table 1, row 21). The MAF protein is a transcription factor containing a leucine zipper that can bind DNA. Because its folding type includes homodimer and heterodimer, it can transactivate target genes to participate in cellular processes. Recently, some studies have demonstrated that c-Maf can interact with c-Myb, downregulate Bcl-2 expression, increase cell death in peripheral CD4 cells [86], and transactivate the tumor suppressor gene* p53* in vitro. The apoptosis of primary cell lines is induced by overexpression of c-Maf via a p53-dependent mechanism [87]. In addition, c-Maf enhances apoptosis through transactivating caspase 6 in peripheral CD8 cells [88]. Taken together, we observed that MAF is closely related to apoptosis.

#### 3.2.19. *NFKB2*

The betweenness of this gene was 8 and its permutation FDR was 0.034 (refer to Table 1, row 22).* NFKB2* encodes a subunit of the transcription factor complex NF-*κ*B. NF-*κ*B significantly functions in regulating the immune response; however, in almost the same manner, it also induces proliferation, inflammation, and regulation of apoptosis [89]. For example, variation in NF-*κ*B activity results in mitochondrial apoptosis after infecting cells with pathological prion proteins. In addition, NFKB2, as a subunit of NF-*κ*B, is involved in the MAPK signaling pathway, which finally regulates proliferation, inflammation, and antiapoptosis. Nevertheless, these hypotheses require further validation.

#### 3.2.20. CALM1

CALM1 (betweenness: 244, permutation FDR: 0.038; refer to Table 1, row 23) is one member of the EF-hand calcium-binding protein family, and its function is regulated by calcium. No report has so far observed a relationship between* CALM1* and apoptosis; however, calcium concentration changes can induce a series of cell biological processes to happen. Because calcium activates the MAPK and JNK signaling pathways, we presume that calcium activates CALM1, which in turn activates downstream gene expression, potentially including apoptosis-related genes, such as* BCL-2*, finally regulating cell survival and apoptosis. In addition, in the STRING analysis, CALM1 has interactions with IQGAP1 and ADCY8. As mentioned above,* IQGAP1* and* ADCY8* have been well characterized to participate in apoptosis. In summary, these arguments all support our results.

#### 3.2.21. *CAMK2B*

The betweenness of this gene was 179 and its permutation FDR was 0.04 (refer to Table 1, row 24). The product of* CAMK2B* is a member of the Ca^2+^/calmodulin-dependent protein kinase subfamily.* CAMK2B* is involved in several pathways, such as the melanogenesis pathway and the neurotrophin signaling pathway. Because* CAMK2B* is downstream of the gene* CALM1*, CALM1 phosphorylates and thereby activates CAMK2B. In addition, CAMK2B is also involved in the Wnt/Ca^2+^ pathway, which dephosphorylates the NFAT transcription factor family, which in turn induces the expression of genes such as CD40L, CTLA-4, and FasL, finally participating in the process of cell fate decision. Recently, research has demonstrated that CAMK2B protects neurons from homocysteine-induced apoptosis with the involvement of the HIF-1*α* signal pathway [90].

#### 3.2.22. *CBLB*

CBLB (betweenness: 5, permutation FDR: 0.042; refer to Table 1, row 25) is an ubiquitin ligase. CBLB can ubiquitinate other proteins to influence biological processes. Cbl-b contributes to the apoptosis induced by the chemotherapy in rat basophilic leukemia cells through inhibiting PI3K/Akt activation and increasing MEK/ERK activation [91]. Downregulating* Cbl-b* by shRNA resulted in strongly activating ERK, JNK, and p38 MAPK [92] and upregulating DR4 and DR5 in the presence of bufalin in MDA-MB-231 and MCF-7 cells. Moreover, the ubiquitin ligase Cbl-b negatively regulates the PI3K/Akt pathway. Therefore, we presume that Cbl-b indirectly mediates cell survival and apoptosis, which requires further experimental exploration.

#### 3.2.23. *JUN*

The betweenness of this gene was 211 and its permutation FDR was 0.044 (refer to Table 1, row 26). JUN regulates gene expression by interacting directly with target DNA sequences. JNK/P38 MAP kinase pathway is crucial in regulating apoptosis, proliferation, differentiation, and inflammation. JUN can be activated by JNK, and JNK in turn transactivates downstream gene expression to perform these functions. However, the details of this regulatory mechanism require further research.

## 4. Conclusion

This contribution attempted to provide a better comprehension of apoptosis by identifying novel apoptosis-related genes. An existing computational method was applied with a weighted graph, constructed by protein-protein interaction information, to search for possible genes related to apoptosis. The analyses of the obtained genes further suggest that they are related to apoptosis.

## Supplementary Material

The Supplementary Material contains two files. In detail, the Supplementary Material I lists 86 human genes that are related to apoptosis; the Supplementary Material II lists 114 candidate genes discovered by our method and their betweenness and permutation FDRs.

## Figures and Tables

**Table 1:** 26 significant candidate genes, their betweenness, and permutation FDRs.

Row number	Ensembl ID	Gene name	Betweenness	Permutation FDR
1	ENSP00000316840	TRAF6	509	<0.002
2	ENSP00000365435	TNFRSF1B	86	<0.002
3	ENSP00000268182	IQGAP1	289	<0.002
4	ENSP00000268171	FURIN	252	<0.002
5	ENSP00000327850	NFATC1	95	<0.002
6	ENSP00000379330	NFATC2	238	<0.002
7	ENSP00000315615	AKAP5	195	<0.002
8	ENSP00000267169	DIABLO	138	<0.002
9	ENSP00000216160	TAB1	120	<0.002
10	ENSP00000246533	CAPNS1	86	<0.002
11	ENSP00000380349	CAPN3	86	<0.002
12	ENSP00000286355	ADCY8	167	0.002
13	ENSP00000382834	NPRL3	83	0.008
14	ENSP00000288840	SMAD6	25	0.014
15	ENSP00000304895	IRS1	238	0.016
16	ENSP00000237596	PKD2	3	0.016
17	ENSP00000296871	CSF2	65	0.02
18	ENSP00000404503	BBC3	8	0.02
19	ENSP00000277541	NOTCH1	249	0.022
20	ENSP00000360683	PTPN1	93	0.024
21	ENSP00000327048	MAF	54	0.026
22	ENSP00000189444	NFKB2	8	0.034
23	ENSP00000349467	CALM1	244	0.038
24	ENSP00000258682	CAMK2B	179	0.04
25	ENSP00000264122	CBLB	5	0.042
26	ENSP00000360266	JUN	211	0.044
