# Supplementary material for: Identifying Novel Candidate Genes Related to Apoptosis from a Protein-Protein Interaction Network
Source: Comput Math Methods Med. 2015 Oct 4;2015:715639. doi: 10.1155/2015/715639 (PMC4620916; doi:10.1155/2015/715639)
Supplement: Supplementary file 1 — The Supplementary Material contains two files. In detail, the Supplementary Material I lists 86 human genes that are related to apoptosis; the Supplementary Material II lists 114 candidate genes discovered by our method and their betweenness and permutation FDRs. [file 715639.f1.zip › Supp-I.pdf]

Supplementary Material I. The 86 human genes that are related to apoptosis.

AIFM1
AKT1
AKT2
AKT3
APAF1
ATM
BAD
BAX
BCL2
BCL2L1
BID
BIRC2
BIRC3
BIRC7
BIRC8
CAPN1
CAPN2
CASP10
CASP12
CASP3
CASP6
CASP7
CASP8
CASP9
CFLAR
CHUK
CSF2RB
CYCS
DFFA
DFFB
ENDOG
FADD
FAS
FASLG
IKBKB
IKBKG
IL1A
IL1B
IL1R1
IL1RAP
IL3
IL3RA

IRAK1
IRAK2
IRAK3
IRAK4
MAP3K14
MYD88
NFKB1
NFKBIA
NGF
NTRK1
PIK3CA
PIK3CB
PIK3CD
PIK3CG
PIK3R1
PIK3R2
PIK3R3
PIK3R5
PPP3CA
PPP3CB
PPP3CC
PPP3R1
PPP3R2
PRKACA
PRKACB
PRKACG
PRKAR1A
PRKAR1B
PRKAR2A
PRKAR2B
PRKX
RELA
RIPK1
TNF
TNFRSF10A
TNFRSF10B
TNFRSF10C
TNFRSF10D
TNFRSF1A
TNFSF10
TP53
TRADD
TRAF2
XIAP
