# Supplementary material for: Identifying Novel Candidate Genes Related to Apoptosis from a Protein-Protein Interaction Network
Source: Comput Math Methods Med. 2015 Oct 4;2015:715639. doi: 10.1155/2015/715639 (PMC4620916; doi:10.1155/2015/715639)
Supplement: Supplementary file 1 — The Supplementary Material contains two files. In detail, the Supplementary Material I lists 86 human genes that are related to apoptosis; the Supplementary Material II lists 114 candidate genes discovered by our method and their betweenness and permutation FDRs. [file 715639.f1.zip › Supp-II.pdf]

Supplementary Material II. 114 candidate genes discovered by our method and their betweenness and permutation FDRs

Ensembl IDs	Gene name	Betweenness	Permutation FDR
ENSP00000365435	TNFRSF1B	86	<0.002
ENSP00000216160	TAB1	120	<0.002
ENSP00000379330	NFATC2	238	<0.002
ENSP00000327850	NFATC1	95	<0.002
ENSP00000268182	IQGAP1	289	<0.002
ENSP00000316840	TRAF6	509	<0.002
ENSP00000268171	FURIN	252	<0.002
ENSP00000267169	DIABLO	138	<0.002
ENSP00000246533	CAPNS1	86	<0.002
ENSP00000380349	CAPN3	86	<0.002
ENSP00000315615	AKAP5	195	<0.002
ENSP00000286355	ADCY8	167	0.002
ENSP00000382834	NPRL3	83	0.008
ENSP00000288840	SMAD6	25	0.014
ENSP00000237596	PKD2	3	0.016
ENSP00000304895	IRS1	238	0.016
ENSP00000296871	CSF2	65	0.02
ENSP00000404503	BBC3	8	0.02
ENSP00000277541	NOTCH1	249	0.022

ENSP00000360683	PTPN1	93	0.024
ENSP00000327048	MAF	54	0.026
ENSP00000189444	NFKB2	8	0.034
ENSP00000349467	CALM1	244	0.038
ENSP00000258682	CAMK2B	179	0.04
ENSP00000264122	CBLB	5	0.042
ENSP00000360266	JUN	211	0.044
ENSP00000355759	PARP1	15	0.056
ENSP00000387699	CREB1	209	0.062
ENSP00000298139	WRN	82	0.066
ENSP00000362994	TRAF1	1	0.066
ENSP00000365016	IRS2	8	0.074
ENSP00000231454	IL5	69	0.078
ENSP00000355537	ACTN2	86	0.088
ENSP00000264246	CD80	7	0.09
ENSP00000267859	BNIP2	64	0.098
ENSP00000310572	PSMC5	29	0.102
ENSP00000298552	TSC1	18	0.114
ENSP00000262367	CREBBP	144	0.114
ENSP00000354621	SMURF1	25	0.128
ENSP00000361125	VEGFA	86	0.134
ENSP00000229794	MAPK14	11	0.14

ENSP00000280357	IL18	58	0.14
ENSP00000269485	TNFRSF11A	17	0.16
ENSP00000353483	MAPK8	209	0.168
ENSP00000401303	SHC1	169	0.17
ENSP00000339191	CAV1	3	0.174
ENSP00000329411	IRF7	2	0.176
ENSP00000361359	CD40	32	0.184
ENSP00000329967	TBK1	2	0.212
ENSP00000306512	IL8	16	0.244
ENSP00000279593	GRIN2B	91	0.26
ENSP00000359206	BTRC	85	0.262
ENSP00000361021	PTEN	96	0.264
ENSP00000229135	IFNG	72	0.268
ENSP00000324890	CD28	12	0.268
ENSP00000344818	UBC	617	0.292
ENSP00000305480	FEN1	3	0.304
ENSP00000226730	IL2	152	0.308
ENSP00000244007	PLCG1	54	0.31
ENSP00000339992	MYB	54	0.31
ENSP00000332049	CD86	5	0.312
ENSP00000269141	CDH2	1	0.328
ENSP00000348986	INS-IGF2	119	0.334

ENSP00000310127	IRF3	2	0.352
ENSP00000210313	PSMD5	11	0.364
ENSP00000371067	JAK2	125	0.364
ENSP00000344352	ATF3	61	0.372
ENSP00000306245	FOS	15	0.38
ENSP00000263923	KDR	9	0.406
ENSP00000330341	SOCS3	1	0.456
ENSP00000344115	CDH5	3	0.462
ENSP00000364898	SYK	5	0.504
ENSP00000356425	UCHL5	18	0.542
ENSP00000343204	JAK1	5	0.586
ENSP00000258743	IL6	7	0.626
ENSP00000338799	IL6ST	3	0.672
ENSP00000303830	INSR	11	0.68
ENSP00000357879	PSMD4	29	0.684
ENSP00000348461	RAC1	172	0.736
ENSP00000231509	NR3C1	5	0.744
ENSP00000368438	PCNA	3	0.766
ENSP00000314458	CDC42	80	0.772
ENSP00000417281	MDM2	168	0.788
ENSP00000309845	HRAS	9	0.828
ENSP00000384675	SOS1	6	0.834

ENSP00000338018	HIF1A	72	0.834
ENSP00000162330	BCAR1	6	0.84
ENSP00000324806	GSK3B	82	0.85
ENSP00000357656	FYN	33	0.854
ENSP00000223023	WASL	10	0.862
ENSP00000340944	PTPN11	19	0.864
ENSP00000354394	STAT1	20	0.886
ENSP00000344456	CTNNB1	262	0.888
ENSP00000268035	IGF1R	6	0.89
ENSP00000011653	CD4	2	0.896
ENSP00000362649	HDAC1	18	0.9
ENSP00000335153	HSP90AA1	5	0.922
ENSP00000261799	PDGFRB	5	0.926
ENSP00000275493	EGFR	252	0.934
ENSP00000364893	ARHGEF7	3	0.94
ENSP00000263253	EP300	278	0.954
ENSP00000341189	PTK2	6	0.958
ENSP00000264033	CBL	30	0.96
ENSP00000302269	VAV1	17	0.962
ENSP00000228872	CDKN1B	15	0.962
ENSP00000262160	SMAD2	25	0.97
ENSP00000356346	PTPRC	2	0.972

ENSP00000267163	RB1	2	0.982
ENSP00000264657	STAT3	131	0.988
ENSP00000227507	CCND1	18	0.994
ENSP00000269571	ERBB2	32	0.998
ENSP00000350941	SRC	12	1
ENSP00000339007	GRB2	60	1
ENSP00000206249	ESR1	12	1
